# Trends in disease-free life expectancy at age 65 in Spain: Diverging patterns by sex, region and disease

**DOI:** 10.1371/journal.pone.0240923

**Published:** 2020-11-11

**Authors:** Pilar Zueras, Elisenda Rentería

**Affiliations:** Centre d’Estudis Demogràfics, Bellaterra, Barcelona, Spain; Sciensano, BELGIUM

## Abstract

Life expectancy in Spain is among the highest in the world. Nevertheless, we do not know if improvements in health conditions at older ages have followed postponements of death. Previous studies in Spain show a stable trend in years lived in ill health in the past. In this paper we investigate changes between 2006, 2012 and 2017 in life expectancy with and without disease at age 65 in Spain and, for the first time, in Spanish regions, which have autonomous powers of health planning, public health and healthcare. Results show that, at the country level, disease-free life expectancy reduced between 2006 and 2017 in Spain. This was explained by an expansion of most diseases except for some cardiovascular and respiratory chronic conditions. However, at the regional level the evolution was different, especially regarding each disease and sex. First, regional differences reduced between 2006 and 2012 but largely widened in 2017, suggesting that not all regions had the same ability to recover after the 2008 financial crisis that caused government cuts to health services. Second, regional analysis also highlighted diverging trends by sex. While men experienced expansion of morbidity in most regions, women experienced a compression in about half of them, ending up with women showing higher disease-free life expectancies than men in 9 out of the 17 regions considered. This study, then, calls attention to the importance of focusing the analysis of health surveillance to more disaggregated levels, more in accordance with the level of health management, as regional trends showed heterogeneity in the prevalence of diseases and different progresses in the relationship between sexes.

## Introduction

Chronic diseases are an important determinant of quality of life, as they cause multiple health impairments, increase the risk of suffering and dying from specific infections, and eventually cause death. Moreover, they drive population health care needs, defining their level of utilization as well as which kinds of medical interventions are required. In a context of increasing life expectancies, as in Spain’s case, it is essential to know if we are increasing or decreasing our time with disease, especially among the older population. Additionally, in the context of the current COVID-19 pandemic, this has become an issue of major concern, as some of these health conditions, in particular hypertension and respiratory and cardiovascular diseases, are associated with a high risk of severe illness [1]. This paper examines changes in life expectancies with and without disease at age 65 for several chronic health conditions by sex in Spain with a regional approach.

In the literature there is an open debate on how increasing life expectancies (LE) have been accompanied, or not, by improvements in health conditions [2–7]. Measuring healthy life expectancies is an ideal instrument for analyzing the evolution of this phenomena, as they take into account morbidity and mortality at the same time by estimating the time that individuals live with a certain condition, disability or disease [8–10]. Most research on health trends draws on disability measures to assess whether there has been an expansion or compression in morbidity based on different possible scenarios of morbidity onset and death occurrence [5,6,11–14]. Fewer studies focus on chronic diseases; however, their consideration in the framework of the analysis of compression of morbidity is crucial. On the one hand, chronic diseases dominate deaths and have a physiological and psychosocial burden on people’s lives and on medication and health care costs of both individuals and societies [7,14]. On the other hand, chronic health conditions may affect the incidence, severity and fatality risk of virulent infections [15], which in the case of epidemics will have an impact on disease-free life expectancies in the older population.

Spain has one of the highest life expectancies in the world, with 80.4 years for men and 85.7 years for women in 2017 [16], but research has not significantly deepened in the study of health expectancies, especially in recent years. More specifically, the analysis of chronic diseases, and their evolution as years of life lived with and without disease, has barely been explored in the case of the older population in Spain [17]. In addition, Spain has a health system that is not centralized, and each region (Autonomous Community, AC) has autonomy regarding health planning, public health and healthcare. Therefore, differences in health expectancies across regions could derive from inequalities in health management in each region, showing, for example, how each region has faced and recovered from the 2008 financial crisis that caused severe cuts in public health expenditures [18].

Previous studies have shown different results on trends in healthy life expectancies in Spain, most of them based on the analysis of disability prevalence. Researchers [13] show a compression of the number of years lived with disability for both men and women before 1999; however, the Ehemu group [5] show a much more stable trend for the subsequent period (1995–2003) when they take into account only people aged 65 years and older. Both studies confirm that women spent more years with disability than men, as seen in other countries [10]. Since 2006, Eurostat has published healthy life expectancies for European countries annually, including Spain, and they show the same trend of compression of morbidity observed before: disability-free life expectancy at birth has continued to increase since 2006; however, this is not so clear at age 65, as the estimate is very stable, and only since 2015 has there been a small improvement in the number of years lived without disabilities.

Few studies analyze the prevalence of recent trends in major chronic diseases and cardiovascular risk factors in Spain and Spanish regions. Walter and colleagues [17] examined the incidence of chronic diseases at the country level and found an expansion of cancer and cardio- and cerebrovascular morbidity in LE at age 50 in 1997–2000 and 2007–10, with evidence of compression only for cerebrovascular disease among women and for lung cancer among men. Cancer and cardiovascular morbidity can also expand as a result of both the increasing survivorship of those who had a heart attack or cancer, and improving early diagnosis and screening [7]. Studies carried out in 2000–09 found relatively low geographical variability of the prevalence of cardiovascular risk factors in the population aged 35–74, with higher accumulation and prevalence observed in the southern regions of Andalusia, Extremadura and the Canary Islands [19].

If we look deeper into the variation between Spanish regions (in a regional study about disease-free life expectancy and life expectancy without mobility limitations), Solé-Auró [20] found an expansion of morbidity in Catalonia from 1994 to 2011. Regional variations in healthy life expectancy (based on activity limitation) between 2007 and 2011 did not reveal a consistent geographical pattern, but life expectancy in good health was lower in the north-western ACs and some Mediterranean ACs [12]. In general, studies show a strong association between quantity and quality of life [6]. However, in 1999, people in Spanish regions with longer LE were not always those with healthier lives, and the geographical variability in disability-free life expectancy was higher than in LE [21]. A study found that the main factors behind those differences were social and economic circumstances rather than health resources [12]. On the contrary, other findings [22] demonstrate that per capita income and health resources, together with decentralization of the health system—measured as a proportion of sub-national health expenditure—were associated with improvements in LE between 1992 and 2003.

Regarding the effects of the economic crisis on the health status of the population, it is true that health and social services budgets were reduced between 2011 and 2013 as a result of the financial crisis. Also, regions addressed austerity measures differently, as observed in co-payment for drugs, privatization or reduction of health services, staff costs, and cutbacks in dependency support for people with disabilities [18,23]. Despite this, during the first years of the crisis, mortality in Spain declined at a higher rate than before, in particular among the low socioeconomic group [24]. This paradoxical result is in concordance with prior studies that found a relationship between economic recessions and a subsequent decrease in mortality rates, at least momentarily [25–28]. However, according to Eurostat data, trends of disability-free life expectancy at age 65 did not show an improvement until 2015. Less is known about trends in lifespan at age 65 spent with and without chronic diseases and health conditions, and how these health indicators vary across regions. Improving the prevention, detection and management of chronic diseases has become a priority in Europe [29] and a challenge for Spain, which was forecasted to be at the forefront of longevity by 2040 [30].

The aim of this paper is to estimate the change between 2006, 2012 and 2017 in life expectancy with and without disease at age 65 in Spain and Spanish regions (ACs). We focus on the prevalence of 10 specific chronic diseases and health conditions: cancer, stroke, myocardial infarction, heart disease, diabetes, hypertension, high cholesterol, chronic back pain, asthma and chronic obstructive pulmonary disease (COPD). By taking a regional approach to life expectancies without these diseases, we aim to better understand the possible trends of compression or expansion of morbidity among those aged 65 years and older and their relationship with changes that have occurred, especially during the period under study, due to the long and harsh economic crisis that Spain faced between 2008 and 2013.

## Materials and methods

### Study design and sample size

We conducted a cross-sectional study using life tables and disease prevalence rates by sex and age group. Data from life tables in Spain come from the National Institute of Statistics of Spain (INE as per its Spanish acronym) and prevalence rates of disease come from the National Health Surveys (ENS as per its Spanish acronym) of 2006, 2012 and 2017, the latest available. The ENS samples are representative of the population at the Autonomous Community (AC) level. We analyzed data for adults aged 65 and older and used 80 years and over as the final age interval in the life table. The study was performed at national and regional levels. In Spain, there are 19 ACs, 15 in the Iberian Peninsula (Andalusia, Aragon, Asturias, Basque Country, Cantabria, Catalonia, Castile-Leon, Castile-La Manche, Madrid, Extremadura, Galicia, La Rioja, Murcia, Navarre and Valencia), plus the Balearic Islands in the Mediterranean Sea, the Canary Islands in the Atlantic Ocean, and Ceuta and Melilla, which are two autonomous cities on the African coast in the strait of Gibraltar. Regarding the quality of response of the ENS, in 2006 96% of all theoretical households were attained, considered as the response rate. This differs by region, but for all of them, this rate was above 90% [31]. In 2012, the response rate was 89.6%, distributed unevenly, with La Rioja showing 98.3% and Ceuta 71% [32]. In 2017, the response rate was lower, with 72.2% of the surveyable households; Melilla had the highest rate with 82.2%, and Madrid showed the lowest rate with 63.8% [33].

Once we had estimated disease prevalence at the AC level in Spain, we encountered problems with data availability for some regions, as they are too small to report information for each disease. Therefore, we had to exclude Ceuta and Melilla. Our sample includes a total of 20,455 individuals aged 65 and older who were respondents in one of the ENS collections: 2006 (n = 7,717, 64.0% women), 2012 (n = 5,815, 62.3% women) or 2017 (n = 6,923, 69.4% women).

### Health variables

We chose to examine changes in cancers and cardio- and cerebrovascular diseases, major chronic diseases that were among the five leading causes of death in 2006–2016 in Spain [34], considering the following four indicators: cancer, stroke, myocardial infarction and heart disease. Additionally, we considered three health conditions that are also cardiovascular risk factors: hypertension, diabetes and high cholesterol. We also included chronic low back and neck pain, which became the main contributor to disability-adjusted life years in 2016, according to the Global Burden of Disease study for Spain [34]. Moreover, we examined asthma and COPD, the two most common respiratory diseases, which are not only leading causes of mortality and burden of disease [34] but also the main predictive factor for severity and intensive care unit admission in case of illness with COVID-19 [1]. In order to keep concordance between years, we could not use other relevant diseases that cause impairment and loss of function that were reported in each database. For example, arthrosis was excluded from the analysis, as the definition changed from 2012 to 2017. However, we could include stroke and COPD despite the change in wording between surveys. Also, the data did not distinguish by type of cancer. The following set of questions addresses the presence of these health conditions: (1) Have you ever suffered from ‘this specific health condition’? (2) Have you had it in the last 12 months? (3) Has a doctor told you that you have it? We considered that individuals have the health condition when they answered the second and third questions in the affirmative.

The reporting of health conditions could be biased by uneven health care access, but there are no differences across ACs in the country, nor are there limitations to diagnosis. The public healthcare in Spain is practically universal, attending 99.9% of the total population [35]. In 2008, the law changed, reducing full access for undocumented immigrants (except for emergency services and maternal and child care), but it is difficult to measure how many people were affected, as not all the regions followed the law equally [36], and we can expect that they are underrepresented in the surveys we are using here. Yet, although universality does not mean full access to healthcare, previous research shows that the inefficiencies in the provision of public health care in Spain are more related to long waiting lists and difficulty paying for additional services not covered by public healthcare (copayment of medication prescriptions and access to dentists). Access to general practitioners or specialists, who would be diagnosing the condition or disease measured here, does not show inequalities by region or socioeconomic status [37]. In fact, when comparing European countries, Spain was ranked as the country with the fifth lowest percentage of unmet needs for a medical examination or treatment due to costs, distance to travel or waiting times, based on a Eurostat Database using EU-SILC data [35].

When conducting the regional analysis of HLE by disease, counts for some conditions, such as cancer, stroke, myocardial infarction, heart disease, asthma and COPD, were too small in some ACs. Therefore, we grouped stroke, myocardial infarction and heart disease in a single category called cardiovascular diseases (CVD) and asthma and COPD as respiratory diseases, and we had to drop cancer from the AC analysis.

### Method

To estimate life expectancies with disease and disease-free life expectancies, we used the Sullivan method [38]. This is a method that estimates the number of expected years that an individual can live at a certain age in a healthy or unhealthy state, given that the individual experiences the same prevalence rates of disease as the population in that year. The estimation is done by using prevalence data on each of the health conditions chosen by age groups (5-year age groups) and sex, applied to an abridged life table.

We calculated life expectancy with disease at age 65 (LE_w_d_) as follows:

$${LE}_{w\_d}^{x}=\frac{\sum_{i=x}^{w}L_{i}D_{i}}{l_{x}}$$ (1)

where L_i_ corresponded to the number of life-years lived between exact ages (x, x+n), l_x_ referred to the number of survivors at age x (in this case 65) and D_i_ was the proportion of people living with the selected disease between exact ages (x, x+n).

Finally, we estimated the measure of healthy life expectancy (HLE) as the time expected to live without any of these heath conditions and diseases. This measure was calculated in a similar way as the previous one:

$${HLE}^{x}=\frac{\sum_{i=x}^{w}L_{i}({1-TD}_{i})}{l_{x}}$$ (2)

where TD_i_ referred to the proportion of people with at least one of the diseases or health conditions considered here with the exact ages (x, x+n).

## Results

### Evolution of life expectancy with and without disease at the country level

**Fig 1:**
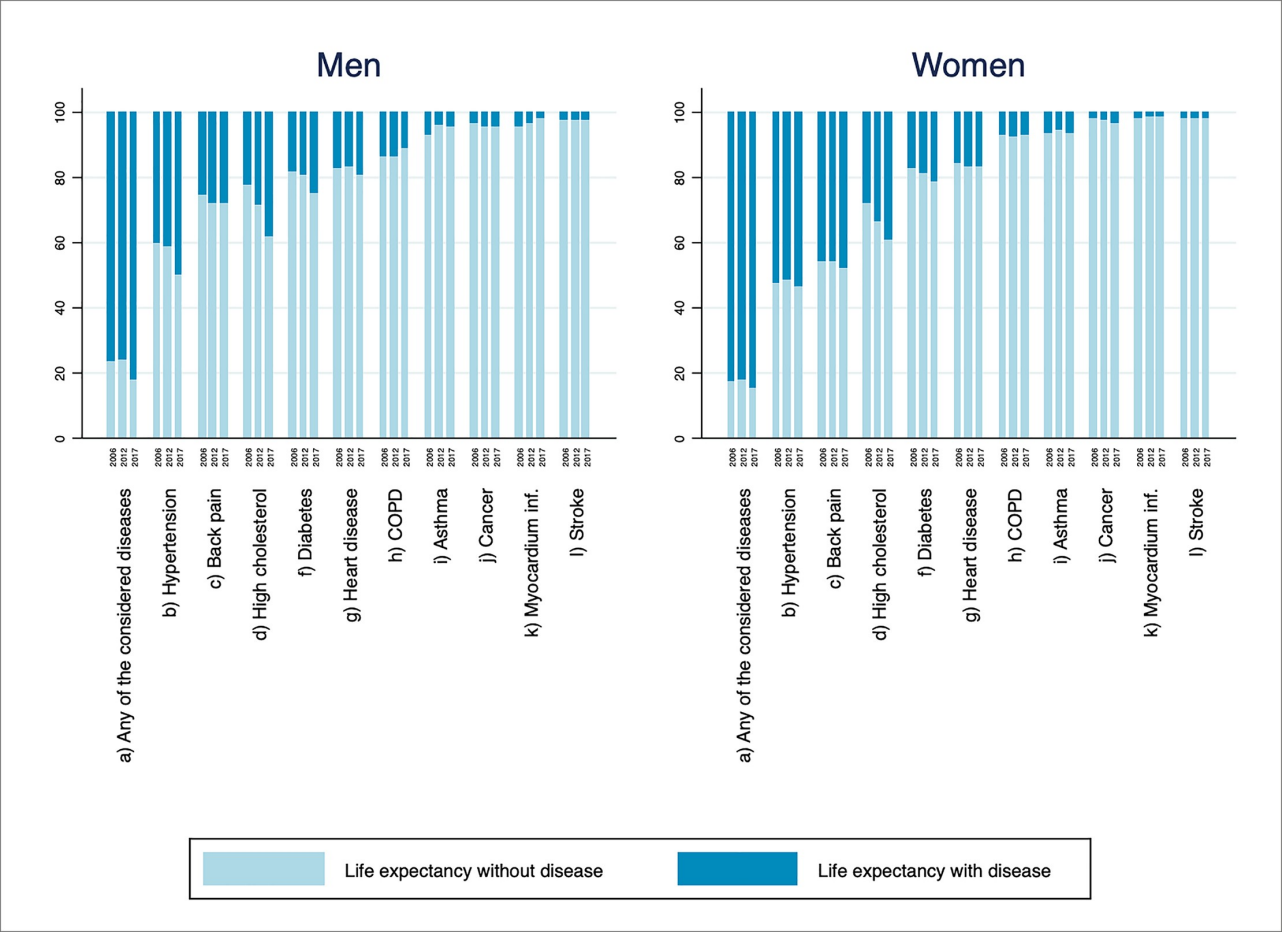
Percentage of expected length of life lived with and without disease at age 65 for Spain in 2006, 2012 and 2017. ‘Any of the considered diseases’ refers to life expectancy with at least one condition or without any of the diseases considered. Source: Authors’ calculations.

We first display the evolution of life expectancies with and without disease over the 3 years of observation for Spain. We use ‘disease-free’ and ‘healthy’ life expectancy (HLE) indistinctly to refer to life expectancy without any disease considered in this study. Fig 1 presents the percentage of LE that is spent free of disease, jointly with the percentage spent with at least one of the health conditions, and separately for each one of the conditions under study (asthma, back pain, COPD, cancer, high cholesterol, diabetes, heart disease, hypertension, myocardial infarction and stroke). The complementary S1 Table provides years spent with each disease together with LE at age 65 by sex, remaining years with and without disease and its proportion. Even though women had the highest LE throughout the period (about four more years of LE at age 65 than men), the percentage of remaining healthy life of men was longer in all years under study. Men’s advantage is not only in relative terms; they enjoyed almost half a healthy year more than women in 2006 and 2012, while in 2017 the number of years expected to live without disease was slightly higher for women. However, that represented only 15.2% of their LE, compared to 17.8% of men’s LE.

We observe that the proportion of LE with disease increased for both sexes in 2017, especially among men. While in 2006 and 2012 men spent around 76% of their LE after 65 years old with at least one disease or health condition, this has worsened in 2017, increasing to 82%. Time trends were similar across sex, showing a small amelioration of HLE in 2012 and a decrease in 2017. Specifically, the remaining time without disease of men in 2006 was 23.5% out of 17.7 years of LE at age 65, which is a HLE of 4.16 years. In 2012, this percentage rose slightly to 23.9% (4.43 years) and decreased to 17.8% (3.41 years) in 2017. Women followed a similar trend but with a smaller decline in 2017, which contributed to narrowing the sex gap observed in the previous years. The HLE of women in 2006 was 17.0% of their remaining life at age 65 (21.7 years), which is a HLE of 3.69 years. It improved to 17.5% (equivalent to 3.93 of 22.4 years) in 2012 and worsened in 2017 to 15.2%, only 3.50 years free of any disease considered, slightly above the HLE of men.

When looking at each disease separately, we see that hypertension is the condition that older people in Spain are going to spend more time with, representing more than 50% of the LE for women and more than 40% for men, closely followed by back pain in the case of women (more than 45% across the 3 years). In the case of men, high cholesterol is the second most prevalent health condition and has percentages similar to those of women. This is a condition that has increased steeply from 2006 to 2017, going from 22% to 38% of LE living with high cholesterol in men, and 28% to 39% in women, meaning between 4.0 and 7.3 of the remaining years of life for men and between 6.1 and 9.0 for women. In fact, the percentage of LE has increased for most of the diseases (back pain, cancer, high cholesterol, diabetes, heart disease for women, hypertension for men); some had a slight improvement until 2012, but deteriorated between 2012 and 2017 (asthma and heart disease for men, and hypertension and myocardial infarction for women). There is only one disease for women (stroke) and four diseases for men (asthma, COPD, stroke and myocardial infarction) whose percentages of LE with the disease have reduced across the three years observed. In the case of stroke and myocardial infarction, their impact is lower than 4% of LE (less than 1 year of LE).

### Evolution of healthy life expectancy by region

Table 1 presents regional variation of HLE by sex and over time by displaying the number of years of LE at age 65 spent free of any of the chronic conditions considered, and the annualized growth of the percentage of years of HLE, by AC and sex in the two periods under study. Results for 2006 reveal a north-south pattern where, with few exceptions, northern regions (Aragon, Basque Country, Cantabria, Castile-León, Catalonia, La Rioja, Madrid, Navarre, Valencia and Balearic Islands) have more years of HLE for both men and women (except La Rioja and Navarre). In the case of men, this pattern blurs in 2012, driven by an improvement of most western and southern regions (Andalusia, Asturias, Castile-La Manche, Extremadura, Galicia and Murcia) with lower HLE and the opposite trend for some ACs that had higher HLE. However, in 2017, there was a decline of healthy years in all ACs except the Balearic Islands, Castile-León and La Rioja. In the case of women, the north-south geographical pattern is less evident in 2006, but it is more visible in 2012 and 2017, showing that in most northeastern regions (Aragon, Basque Country, Cantabria, Castile-León, La Rioja, Madrid, Navarre, Valencia and Balearic Islands) women enjoyed more healthy years than the country average, with the exception of Catalonia, where women’s HLE was about 5 years in 2006 and 2012 and declined to 3 in 2017. In general, for both men and women, regional differences increased by 1.1 years between 2006 and 2017. In the case of men regional differences increased from 3.4 to 4.5, despite reducing to 2.4 in 2012, and in the case of women the increase was gradual (3.2, 4.0 and 4.3). The region that experienced the largest improvements, in terms of numbers of HLE years at age 65 across the time span examined, was La Rioja. Even with a small reduction in years of HLE among men in 2012, they added 0.9 years and women 2.2 between 2006 and 2017; it became the AC with the highest HLE at age 65 for men (6.5 years) and women (5.4 years) together with the Balearic Islands. On the other end, Galician men and women had the lowest HLE in 2017 (2.0 and 1.1 years, respectively).

**Table 1:** Disease-free life expectancy (HLE) with 95% confidence intervals and annual percentage change (APC) in the percentage of HLE at age 65, by sex and region in Spain 2006, 2012 and 2017.

Autonomous Community	2006			2012			2017			APC of the % of years of HLE		
	HLE	CI	% LE	HLE	CI	% LE	HLE	CI	% LE	2006–2012	2012–2017	2006–2017
**Men**												
Andalusia	3.04	(2.86–3.22)	18.15	4.21	(3.97–4.45)	24.15	3.75	(3.55–3.95)	20.59	4.87	-3.14	1.15
Aragon	5.38	(5.12–5.64)	29.90	5.03	(4.59–5.47)	26.87	2.62	(2.42–2.82)	13.63	-1.77	-12.70	-6.90
Asturias	2.46	(2.24–2.68)	14.31	4.77	(4.29–5.26)	26.70	3.16	(2.81–3.51)	17.02	10.95	-8.61	1.59
Balearic Islands	4.33	(3.93–4.74)	24.77	4.00	(3.52–4.47)	21.78	4.20	(3.83–4.57)	21.89	-2.12	0.10	-1.12
Basque country	4.66	(4.24–5.07)	26.32	4.39	(4.03–4.75)	23.57	3.37	(3.14–3.60)	17.57	-1.82	-5.71	-3.61
Canary Islands	5.87	(5.22–6.52)	34.08	4.81	(4.33–5.28)	26.40	4.28	(3.95–4.61)	22.56	-4.17	-3.10	-3.68
Cantabria	5.85	(5.41–6.29)	33.32	5.65	(5.11–6.20)	30.81	3.91	(3.53–4.30)	20.62	-1.30	-7.72	-4.27
Castile-La Manche	4.07	(3.78–4.35)	22.11	5.49	(4.89–6.10)	28.96	2.89	(2.72–3.05)	15.15	4.61	-12.15	-3.38
Castile-Leon	4.29	(3.99–4.59)	22.89	4.23	(3.96–4.50)	21.91	4.52	(4.19–4.86)	22.90	-0.73	0.89	0.00
Catalonia	4.57	(4.30–4.85)	25.70	3.72	(3.51–3.94)	20.02	3.56	(3.39–3.73)	18.58	-4.08	-1.47	-2.90
Extremadura	3.57	(3.25–3.89)	20.66	4.50	(4.12–4.88)	25.27	3.60	(3.29–3.91)	19.50	3.42	-5.06	-0.53
Galicia	3.46	(3.31–3.60)	19.24	3.88		20.65	2.04	(1.94–2.13)	10.57	1.18	-12.53	-5.30
La Rioja	5.61	(4.99–6.22)	30.92	5.30		28.73	6.53	(5.82–7.24)	32.97	-1.22	2.79	0.58
Madrid	4.53	(4.21–4.85)	24.88	5.00		25.63	3.68	(3.48–3.89)	18.35	0.49	-6.46	-2.73
Murcia	2.85	(2.64–3.05)	16.45	3.22		17.84	2.91	(2.60–3.21)	15.64	1.36	-2.60	-0.46
Navarre	4.53	(4.19–4.88)	24.65	3.81		19.88	2.92	(2.71–3.14)	15.15	-3.52	-5.29	-4.33
Valencia	4.56	(4.22–4.89)	26.11	4.48		24.45	2.56	(2.42–2.70)	13.63	-1.09	-11.03	-5.74
**Women**												
Andalusia	3.38	(3.25–3.51)	16.52	2.20		10.41	3.08	(2.97–3.18)	14.17	-7.42	6.37	-1.39
Aragon	4.07	(3.92–4.21)	18.62	3.98		17.65	4.80	(4.53–5.08)	20.71	-0.89	3.26	0.97
Asturias	2.71	(2.57–2.85)	12.44	2.18		9.67	1.93	(1.83–2.02)	8.42	-4.11	-2.73	-3.49
Balearic Islands	4.31	(4.09–4.53)	20.23	4.67		21.30	5.39	(4.97–5.81)	23.77	0.86	2.21	1.47
Basque country	3.70	(3.42–3.99)	16.48	4.12		17.83	4.75	(4.47–5.04)	20.20	1.32	2.53	1.87
Canary Islands	2.86	(2.69–3.03)	13.78	2.60		12.03	3.02	(2.81–3.22)	13.52	-2.24	2.37	-0.17
Cantabria	4.83	(4.60–5.06)	21.64	5.69		24.79	3.54	(3.34–3.73)	15.03	2.29	-9.53	-3.26
Castile-La Manche	3.28	(3.08–3.49)	15.18	2.80		12.52	3.12	(2.93–3.31)	13.73	-3.16	1.86	-0.91
Castile-León	4.36	(4.14–4.58)	19.18	3.48		14.88	5.13	(4.83–5.42)	21.52	-4.14	7.66	1.05
Catalonia	4.99	(4.74–5.24)	22.82	5.11		22.63	3.02	(2.90–3.15)	13.06	-0.14	-10.41	-4.94
Extremadura	1.77	(1.69–1.84)	8.34	3.22		14.71	3.36	(3.14–3.59)	15.11	9.93	0.55	5.56
Galicia	2.78	(2.71–2.85)	12.65	2.89		12.73	1.08	(1.04–1.11)	4.63	0.10	-18.32	-8.74
La Rioja	3.23	(3.02–3.44)	14.26	5.13		22.21	5.38	(4.91–5.85)	22.81	7.66	0.54	4.36
Madrid	3.59	(3.42–3.77)	16.08	6.18		26.38	4.02	(3.85–4.19)	16.83	8.61	-8.60	0.42
Murcia	2.13	(2.05–2.22)	10.32	3.34		15.42	3.16	(2.99–3.34)	14.28	6.92	-1.52	3.00
Navarre	3.08	(2.91–3.25)	13.51	5.46		23.21	3.80	(3.46–4.13)	16.16	9.44	-6.98	1.65
Valencia	3.67	(3.47–3.86)	17.43	4.23		19.29	4.19	(3.99–4.39)	18.70	1.70	-0.61	0.64

Note: Diseases considered include asthma, back pain, cancer, COPD, diabetes, heart disease, high cholesterol, hypertension, myocardial infarction and stroke.

Source: Authors’ calculations.

As observed for the whole country, the remaining years of LE free of disease were higher among men than women in most regions in 2006 (except Andalusia, Asturias, Castile-León and Catalonia) and 2012 (except in Balearic Islands, Catalonia, Madrid, Murcia and Navarra). However, in 2017, women performed better than men in nine of the seventeen ACs analyzed (Aragon, Balearic Islands, Basque Country, Castile-La Manche, Castile-León, Madrid, Murcia, Navarre and Valencia). Women’s greater number of HLE years results from men’s worsening outcomes rather than from an improvement in years of HLE among women between 2006 and 2017, which was observed in all but six ACs (Andalusia, Asturias, Cantabria, Castile-La Manche, Catalonia and Galicia). Over time, men’s advantage in HLE decreased. The largest sex gap in the number of HLE years was found in 2006, showing an advantage of 3.0 healthy years for men in the Canary Islands; this gap reduced to 2.7 in Castile-La Manche in 2012 and to 1.3 in 2017 again in the Canary Islands. Conversely, the advantage for women found in some ACs increased throughout the period from 0.4 healthy years in 2006 in Catalonia, to 1.7 in 2012 in Navarre and 2.2 in Aragon in 2017. Thus, not only did the number of regions where women performed better than men increase between 2006 and 2017, but also the largest sex difference in the number of healthy years found across ACs, which had shown an advantage for men in 2006, became positive for women in 2017.

To see if there has been compression or expansion of morbidity, we need to look at the evolution of HLE relative to LE, i.e. HLE as a percentage of LE. The last columns in Table 1 summarize the annualized growth of the percentage of LE lived free of any of these diseases. Regarding men, the decrease of HLE as a percentage of LE occurred in the majority of ACs, both in periods 2006–2012 and 2012–2017, resulting in only four ACs (Andalusia, Asturias, Castile-León and La Rioja) experiencing a real compression of morbidity from 2006 to 2017, which represented less than a 1.6 annual percentage change (APC) for each AC. In the case of women, the improvement of HLE as a percentage of LE occurred in most ACs in periods 2006–2012 and 2012–2017. Overall, for the whole period under study (2006–2017), in 10 out of 17 ACs (Aragon, Balearic Islands, Basque Country, Castile-León, Extremadura, La Rioja, Madrid, Murcia, Navarre and Valencia) women experienced a compression in morbidity, which was highest in Extremadura (APC of 5.6%) and lowest in Madrid (APC of 0.4%).

### Regional analysis of the evolution of life expectancy with presence of disease

**Fig 2:**
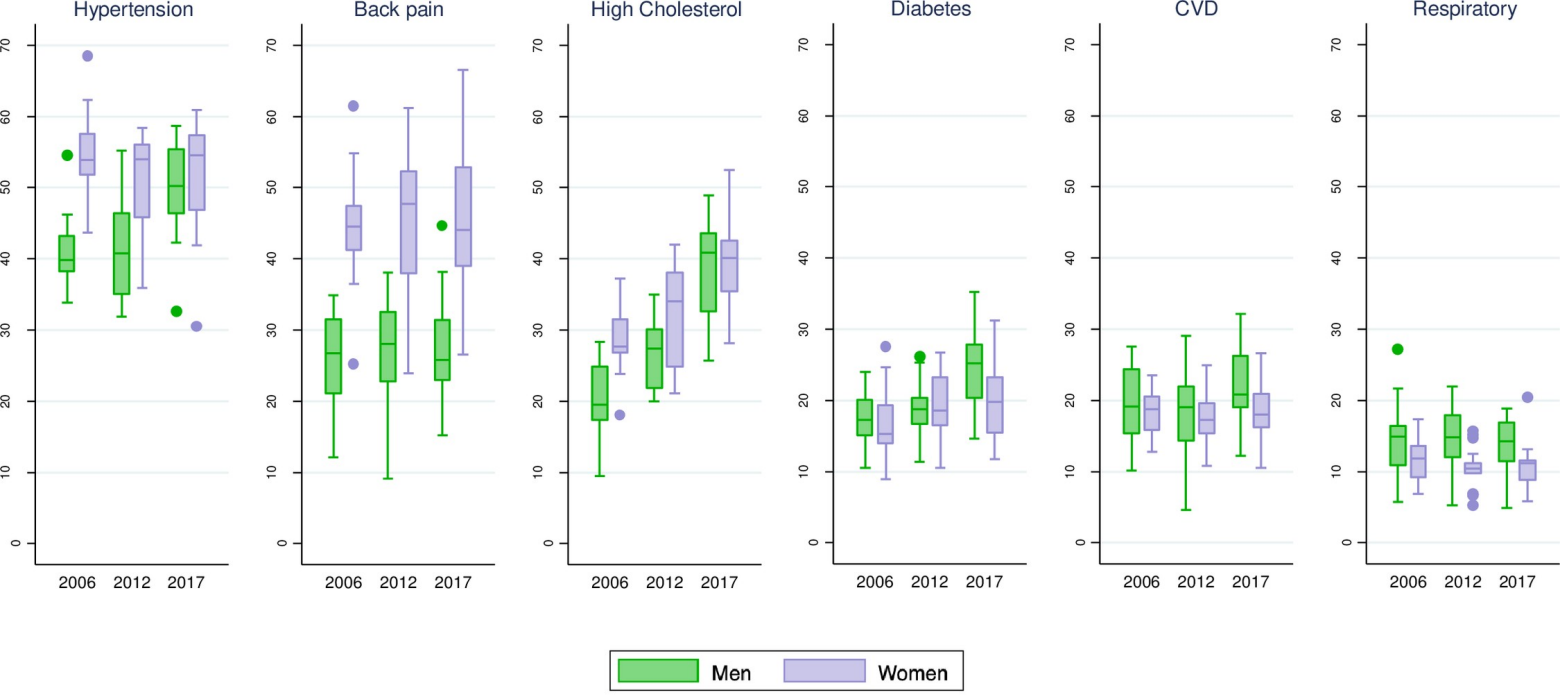
Distribution of the percentage of life expectancy with disease over Spanish Autonomous Communities, by disease and sex in 2006, 2012 and 2017. Source: Authors’ calculations.

When analyzing the evolution of the time spent with diseases separately by AC (Fig 2), we see convergence or divergence depending on the disease or health condition under study and the sex. For both men and women, high cholesterol and diabetes (to a lesser extent among women), and hypertension for men show a general increase in the percentage of LE with disease. In the case of women, the evolution of LE with back pain and high cholesterol shows there is high heterogeneity by AC. Regarding CVD and respiratory disease, there are always more ACs where men have a tendency to decrease the percentage of LE with disease, although the median of all ACs would not go in this direction. As in the analysis of HLE (disease-free LE), we can see that, in all years and most ACs, the percentage of LE with a disease is higher in women than in men in hypertension, high cholesterol and, particularly, back pain (S2 Table). But the opposite is true for men in the case of CVD, respiratory disease and, especially in 2017, diabetes. Furthermore, at the end of the period observed, the indicators of both sexes converged in regard to hypertension, high cholesterol, and to a lesser extent diabetes, CVD and respiratory disease; this resulted from the combination of an increase in the prevalence of diseases in men with the amelioration of women in some ACs.

## Discussion

The main contribution of this paper is that the regional analysis shows that the national estimates usually hide a great heterogeneity in regional trends of the prevalence of diseases and different progresses in the relationship between sexes. We are showing the most recent changes in life expectancies without disease for Spain and its ACs, including different health conditions, that have revealed that the compression or expansion of morbidity depends greatly on the region, sex and health condition.

Regarding changes over time in HLE at age 65, including LE with and without diseases, by sex at the national level, we found that for both men and women HLE reduced between 2006 and 2017 in Spain. This means that the fraction of remaining life spent with at least one of the health conditions considered increased to 82% and 85% of the LE in 2017 for men and women, respectively, meaning 15.7 and 19.5 years with disease. In line with recent research, we found an expansion of most of those diseases [17], with the exception of myocardial infarction and COPD, among men, and stroke for both sexes. For those fatal diseases, this trend could be due to either increased mortality or decreased incidence. However, the mortality rate of myocardial infarction has declined, which implies lower occurrence [39]. Moreover, previous evidence of a slight delay in the onset of CVD and a reduction of incidence of cerebrovascular disease among women [17] suggests that they were the forerunners of the amelioration that we observed among men too. The improvement observed among men in asthma and COPD reflects the evolution of the reduction of smoking among younger cohorts, which was the main risk factor of respiratory and cardiovascular diseases for 1990–2016 in Spain [34]. The small proportion of LE in presence of cancer could be partly an artifact of how the presence of disease was measured—including both the diagnosis of the doctor and having had the condition for the past 12 months—as survivorship may be related to healing.

A traditional north-south geographical pattern arose in 2006 showing better outcomes for the northern areas, in line with previous evidence of disability-free life expectancy [12,19,21,39]. This configuration persisted in the case of women but disappeared among men. In the case of men, this pattern vanished in 2012 due to opposite trends—improvements in regions with lower HLE and deterioration in those with higher HLE—which led to narrowing regional differences. In 2017, differences among men were more pronounced between the western and eastern ACs. Overall, regional differences increased from 2006 to 2017, which could be an effect of the devolution of health system-related powers to the regional level in 2002 [18,40]. This gap reduced between 2006 and 2012 but largely widened in 2017, suggesting that not all regions had the same ability to recover after the financial crisis.

Despite the general expansion of morbidity observed in Spain, especially between 2012 and 2017, trends varied across regions and by sex. The most interesting result of our analysis were the sex differences found in the progression of health expectancy free of disease. While between 2006 and 2017 men showed an expansion of morbidity in all but four ACs, women experienced compression in ten out of the ACs. The fact that populous regions like Andalusia or Catalonia showed an expansion of morbidity over the period could explain that, even with more than half of ACs showing amelioration in the time spent with disease, this is not translated into a decrease in the percentage of LE with disease for the whole country among women. Yet, these opposite trends among men and women are significant in terms of the number of years of LE spent without disease. As expected, and in line with previous research [5,10,13], the number of HLE years was higher among men than women throughout the period in Spain and also in most regions in 2006 and 2012. However, in 2017 the number of years expected to live without disease was higher among women than men in nine ACs. This remarkable result arose from a slight improvement in the HLE of women along with the worsening conditions of men.

Regarding the evolution of the LE spent with each health condition by AC, we observed diverging patterns. There is a steep increase in the length of life spent with high cholesterol, observed since 2006 in all ACs and in both sexes, being steeper among men. Yet, this could be partially due to changes in the diagnosis of high cholesterol, driven by lower recommended levels of low-density lipoprotein (LDL), revised in 2011 [41] and 2016 [42]. This increase in the prevalence of high cholesterol accompanied by early treatment should have had a positive impact on CVD prevention; however, the time trend evolution of CVD in terms of HLE is very stable, meaning that improvements at the onset of CVD have occurred at a similar rate to that of LE. It is distressing to find a lengthening of LE spent with hypertension and diabetes among the majority of ACs, which, together with high cholesterol, increased more among men than women—a result that was highlighted especially by the regional analysis. This is related to the increasing trends in BMI and obesity in Spain during recent years that have been more pronounced among men than women [43,44] and which reflect the deterioration of positive health habits like a healthy diet or physical activity. On the other hand, the reduction of smoking behavior, which was greater among men than women, had a positive impact on shortening LE with respiratory diseases for both men and women [34]. Regarding back pain, the regional analyses show a gradual expansion in the heterogeneity between regions, although the median level increases between 2006 and 2012 and decreases again in 2017. It is essential to monitor back pain because it is one of the main causes of disability in the world [45]. In Spain’s case, previous research observed a decrease in back pain from 2006 to 2009—but not a decrease in lower back pain alone [46]—and an increase between 2009 and 2012 [47], which is more in accordance with the trend observed until 2012, but not until 2017. Differences in AC trends could be a reflection of various factors, from variations in the distribution of individual characteristics (such as socioeconomic status or personal habits) to divergences in the management of public health, as has been pointed out in previous LE studies [12,48]. More in-depth analysis of these inequalities by AC could help to better explain regional disparities.

The implications of the results discussed here are especially critical in light of the current global health crisis due to COVID-19, which has impacted Spain significantly. A profound study on the prevalence and evolution of chronic diseases and risk factors should help health management institutions prepare for the immediate demand of health services and better protect those in the most vulnerable situations. The increase of some of the risk factors closely related with the severity of illness caused by COVID-19 infection [1,15], such as hypertension and diabetes, which are also highly prevalent among older people, should be of special concern to health authorities.

This study has some limitations to the data and methods used in the analysis. First, this is a cross-sectional analysis and, therefore, it does not measure real transition rates between being healthy and turning ill. Also, the severity of health conditions is unknown, which could hide potential improvements in quality of life if the severity of disease was reduced while the number of years with disease was constant. A more refined approach to capturing such improvements in future research would be to analyze HLE based on an indicator combining the presence of a health condition with good or poor self-perceived health. Second, the Sullivan method to calculate HLE uses cross-sectional life tables and observed prevalence of disease instead of incidence; these data are more rapidly available and ready to use. Despite the limitations of this approach, it is recommended for its simplicity and ease of interpretation, and the estimates of healthy life expectancies obtained with the Sullivan method are similar to those produced by other more accurate methods like multistate methods for longitudinal data [49]. Another limitation of using disease prevalence data is the survival effect. Given that some of the conditions under study are more life-threatening than others, it is not surprising that LE with less fatal diseases like hypertension or back pain is longer than LE with cancer, myocardial infarction or stroke. In addition, due to the lack of information in some or all the survey years, we could not explore trends on other important diseases, for example, arthritis or dementia, which cause notable individual and social burden and costs. Finally, as with most surveys, the ENS sample is selected, as it is based on people living in private households and excludes institutionalized people, who are presumably in worse health conditions, which could bias the HLE outcomes at age 65. However, the proportion of people living in such arrangements is low in Spain: about 7% among those aged 85 to 89 in 2011 and lower at younger ages [50]. Despite moderate variation across ACs and an increased share of older people living in institutions between the two last censuses from 2001 and 2011 (INE), we don’t consider these to be a source of important bias for the analysis, as the population living in households in 2011 (sampled in the ENS2012) ranged between 94% and 98% of the total population aged 65 and over (and varied between 95% and 99% in 2001).

## Conclusions and recommendations

This study found an expansion of morbidity in Spain that hides regional diverging trends between 2006 and 2017. We also observed disparities in HLE trends by sex across regions. One of the most relevant results is that although LE free of disease is higher among men than women at the country level, in several ACs the additional effects of the improvement of women’s HLE and the worsening of men’s over time led to women outperforming men at the end of the period observed. The difference in the evolution of HLE between men and women highlights the importance of further expanding this type of analysis in order to understand if they are a consequence of changes in the composition of the older population by sex, due to diverging trends in health-related behaviors for men and women, or of women having benefited more than men from changes in the provision of health services, for example. It is also worth highlighting that regional differences in HLE decreased between 2006 and 2012 and widened again by 2017. This is important because management of the health system depends on each AC, suggesting differences in their recovery following the financial crisis.

Expanding the number of years living with chronic diseases calls attention to the importance of the care system for older people. In a country such as Spain, where it is mainly informally handled, our results suggest that the care burden of caregivers (mainly middle-aged women) might be increasing. Better health service provisions are usually related to better attention when responding to emergencies and increasing survivorship once the disease has already occurred. However, this kind of intervention causes a delay in the moment of death, increasing the time that the individual spends with the disease. When dealing with chronic diseases, what would contribute to a reduction in morbidity is enhancing prevention of the onset of the disease. Public health services must promote, then, the engagement of individuals in better health habits, such as quitting smoking, exercising and embracing healthy diets, which are all powerful tools for improving individual health.

## Supporting information

S1 Table Life expectancy at age 65 and remaining years with each disease (95% confident intervals) by sex in Spain 2006, 2012 and 2017. (DOCX)

S2 Table Percentage of life expectancy with each disease at age 65 by sex in Spanish Autonomous Communities 2006, 2012 and 2017. (DOCX)
