# Correction: Trends in disease-free life expectancy at age 65 in Spain: Diverging patterns by sex, region and disease

**DOI:** 10.1371/journal.pone.0249115

**Published:** 2021-03-19

**Authors:** Pilar Zueras, Elisenda Rentería

In Table 1, values reporting HLE confidence intervals in 2012 are missing for most regions analyzed in the study. Please see the correct Table 1 here.

**Table 1:** Disease-free life expectancy (HLE) with 95% confidence intervals and annual percentage change (APC) in the percentage of HLE at age 65, by sex and region in Spain 2006, 2012 and 2017.

Autonomous Community		2006							2012							2017							APC of the % of years of HLE		
		HLE	CI					% LE	HLE		CI				% LE	HLE		CI				% LE	2006–2012	2012–2017	2006–2017
**Men**																									
	Andalusia	3.04	(	2.86	-	3.22	)	18.15	4.21	(	3.97	-	4.45	)	24.15	3.75	(	3.55	-	3.95	)	20.59	4.87	-3.14	1.15
	Aragon	5.38	(	5.12	-	5.64	)	29.90	5.03	(	4.59	-	5.47	)	26.87	2.62	(	2.42	-	2.82	)	13.63	-1.77	-12.70	-6.90
	Asturias	2.46	(	2.24	-	2.68	)	14.31	4.77	(	4.29	-	5.26	)	26.70	3.16	(	2.81	-	3.51	)	17.02	10.95	-8.61	1.59
	Balearic Islands	4.33	(	3.93	-	4.74	)	24.77	4.00	(	3.52	-	4.47	)	21.78	4.20	(	3.83	-	4.57	)	21.89	-2.12	0.10	-1.12
	Basque country	4.66	(	4.24	-	5.07	)	26.32	4.39	(	4.03	-	4.75	)	23.57	3.37	(	3.14	-	3.60	)	17.57	-1.82	-5.71	-3.61
	Canary Islands	5.87	(	5.22	-	6.52	)	34.08	4.81	(	4.33	-	5.28	)	26.40	4.28	(	3.95	-	4.61	)	22.56	-4.17	-3.10	-3.68
	Cantabria	5.85	(	5.41	-	6.29	)	33.32	5.65	(	5.11	-	6.20	)	30.81	3.91	(	3.53	-	4.30	)	20.62	-1.30	-7.72	-4.27
	Castile-La Manche	4.07	(	3.78	-	4.35	)	22.11	5.49	(	4.89	-	6.10	)	28.96	2.89	(	2.72	-	3.05	)	15.15	4.61	-12.15	-3.38
	Castile-Leon	4.29	(	3.99	-	4.59	)	22.89	4.23	(	3.96	-	4.50	)	21.91	4.52	(	4.19	-	4.86	)	22.90	-0.73	0.89	0.00
	Catalonia	4.57	(	4.30	-	4.85	)	25.70	3.72	(	3.51	-	3.94	)	20.02	3.56	(	3.39	-	3.73	)	18.58	-4.08	-1.47	-2.90
	Extremadura	3.57	(	3.25	-	3.89	)	20.66	4.50	(	4.12	-	4.88	)	25.27	3.60	(	3.29	-	3.91	)	19.50	3.42	-5.06	-0.53
	Galicia	3.46	(	3.31	-	3.60	)	19.24	3.88	(	3.63	-	4.13	)	20.65	2.04	(	1.94	-	2.13	)	10.57	1.18	-12.53	-5.30
	La Rioja	5.61	(	4.99	-	6.22	)	30.92	5.30	(	4.79	-	5.82	)	28.73	6.53	(	5.82	-	7.24	)	32.97	-1.22	2.79	0.58
	Madrid	4.53	(	4.21	-	4.85	)	24.88	5.00	(	4.65	-	5.34	)	25.63	3.68	(	3.48	-	3.89	)	18.35	0.49	-6.46	-2.73
	Murcia	2.85	(	2.64	-	3.05	)	16.45	3.22	(	2.84	-	3.60	)	17.84	2.91	(	2.60	-	3.21	)	15.64	1.36	-2.60	-0.46
	Navarre	4.53	(	4.19	-	4.88	)	24.65	3.81	(	3.42	-	4.20	)	19.88	2.92	(	2.71	-	3.14	)	15.15	-3.52	-5.29	-4.33
	Valencia	4.56	(	4.22	-	4.89	)	26.11	4.48	(	4.16	-	4.79	)	24.45	2.56	(	2.42	-	2.70	)	13.63	-1.09	-11.03	-5.74
	**Women**																
	Andalusia	3.38	(	3.25	-	3.51	)	16.52	2.20	(	2.12	-	2.28	)	10.41	3.08	(	2.97	-	3.18	)	14.17	-7.42	6.37	-1.39
	Aragon	4.07	(	3.92	-	4.21	)	18.62	3.98	(	3.74	-	4.21	)	17.65	4.80	(	4.53	-	5.08	)	20.71	-0.89	3.26	0.97
	Asturias	2.71	(	2.57	-	2.85	)	12.44	2.18	(	2.06	-	2.29	)	9.67	1.93	(	1.83	-	2.02	)	8.42	-4.11	-2.73	-3.49
	Balearic Islands	4.31	(	4.09	-	4.53	)	20.23	4.67	(	4.31	-	5.03	)	21.30	5.39	(	4.97	-	5.81	)	23.77	0.86	2.21	1.47
	Basque country	3.70	(	3.42	-	3.99	)	16.48	4.12	(	3.85	-	4.38	)	17.83	4.75	(	4.47	-	5.04	)	20.20	1.32	2.53	1.87
	Canary Islands	2.86	(	2.69	-	3.03	)	13.78	2.60	(	2.44	-	2.76	)	12.03	3.02	(	2.81	-	3.22	)	13.52	-2.24	2.37	-0.17
	Cantabria	4.83	(	4.60	-	5.06	)	21.64	5.69	(	5.19	-	6.20	)	24.79	3.54	(	3.34	-	3.73	)	15.03	2.29	-9.53	-3.26
	Castile-La Manche	3.28	(	3.08	-	3.49	)	15.18	2.80	(	2.66	-	2.93	)	12.52	3.12	(	2.93	-	3.31	)	13.73	-3.16	1.86	-0.91
	Castile-León	4.36	(	4.14	-	4.58	)	19.18	3.48	(	3.31	-	3.65	)	14.88	5.13	(	4.83	-	5.42	)	21.52	-4.14	7.66	1.05
	Catalonia	4.99	(	4.74	-	5.24	)	22.82	5.11	(	4.91	-	5.32	)	22.63	3.02	(	2.90	-	3.15	)	13.06	-0.14	-10.41	-4.94
	Extremadura	1.77	(	1.69	-	1.84	)	8.34	3.22	(	2.98	-	3.46	)	14.71	3.36	(	3.14	-	3.59	)	15.11	9.93	0.55	5.56
	Galicia	2.78	(	2.71	-	2.85	)	12.65	2.89	(	2.76	-	3.01	)	12.73	1.08	(	1.04	-	1.11	)	4.63	0.10	-18.32	-8.74
	La Rioja	3.23	(	3.02	-	3.44	)	14.26	5.13	(	4.77	-	5.49	)	22.21	5.38	(	4.91	-	5.85	)	22.81	7.66	0.54	4.36
	Madrid	3.59	(	3.42	-	3.77	)	16.08	6.18	(	5.85	-	6.50	)	26.38	4.02	(	3.85	-	4.19	)	16.83	8.61	-8.60	0.42
	Murcia	2.13	(	2.05	-	2.22	)	10.32	3.34	(	3.04	-	3.64	)	15.42	3.16	(	2.99	-	3.34	)	14.28	6.92	-1.52	3.00
	Navarre	3.08	(	2.91	-	3.25	)	13.51	5.46	(	5.04	-	5.88	)	23.21	3.80	(	3.46	-	4.13	)	16.16	9.44	-6.98	1.65
	Valencia	3.67	(	3.47	-	3.86	)	17.43	4.23	(	3.98	-	4.47	)	19.29	4.19	(	3.99	-	4.39	)	18.70	1.70	-0.61	0.64

Note: Diseases considered include asthma, back pain, cancer, COPD, diabetes, heart disease, high cholesterol, hypertension, myocardial infarction and stroke.

Source: Authors’ calculations.

There are formatting errors in S1 Table and S2 Table. Please view the correct S1 Table and S2 Table below.

## Supporting information

S1 Table Life expectancy at age 65 and remaining years with each disease (95% confident intervals) by sex in Spain 2006, 2012 and 2017. (PDF)

S2 Table Percentage of life expectancy with each disease at age 65 by sex in Spanish Autonomous Communities 2006, 2012 and 2017. (PDF)
