# Supplementary material for: Trends in disease-free life expectancy at age 65 in Spain: Diverging patterns by sex, region and disease
Source: PLoS One. 2020 Nov 11;15(11):e0240923. doi: 10.1371/journal.pone.0240923 (PMC7657566; doi:10.1371/journal.pone.0240923)
Supplement: S1 Table — (DOCX) [file pone.0240923.s001.docx]

Table S1. Life expectancy at age 65 and remaining years with each disease (95% confident intervals) by sex in Spain 2006, 2012 and 2017.

|  | **Men** | | | | | | | | | | | | | | | | | | | | | | | | | | | | | | | | | | | | | | | | | | | | | | | | | | | | | | | | | | | | | | | | | | | | | | | | | | | | | | | | | | | | | | | | | | | |  |  |
| --- | --- | --- | --- | --- | --- | --- | --- | --- | --- | --- | --- | --- | --- | --- | --- | --- | --- | --- | --- | --- | --- | --- | --- | --- | --- | --- | --- | --- | --- | --- | --- | --- | --- | --- | --- | --- | --- | --- | --- | --- | --- | --- | --- | --- | --- | --- | --- | --- | --- | --- | --- | --- | --- | --- | --- | --- | --- | --- | --- | --- | --- | --- | --- | --- | --- | --- | --- | --- | --- | --- | --- | --- | --- | --- | --- | --- | --- | --- | --- | --- | --- | --- | --- | --- | --- | --- | --- | --- | --- | --- | --- | --- | --- | --- |
| **Year** | **2006** | | | | | | | | | | | | | | | | | | | | | |  | | **2012** | | | | | | | | | | | | | | | | | | | | | | | | | | | | | | | | | |  | | | **2017** | | | | | | | | | | | | | | | | | | | | | | | | | | | | | | |  |  |
| **Life expectancy 65+** | 17.70 | |  | | | 100% | | | | | | | |  | | | | | | | | | | | 18.51 | | | |  | | | | | | | | | 100% | | | | | | | | |  | | | | | | | | | | | |  | | | | | | | 19.12 | | | | | |  | | | 100% | | | | | | | | | | | |  | |  | | | |  |  |
| **HLE (disease-free)** | 4.16 | |  | | | 23.5% | | | | | | | |  | | | | | | | | | | | 4.43 | | | |  | | | | | | | | | 23.9% | | | | | | | | |  | | | | | | | | | | | |  | | | | | | | 3.41 | | | | | |  | | | 17.8% | | | | | | | | | | | |  | |  | | | |  |  |
| **LE with disease** | 13.54 | |  | | | 76.5% | | | | | | | |  | | | | | | | | | | | 14.08 | | | |  | | | | | | | | | 76.1% | | | | | | | | |  | | | | | | | | | | | |  | | | | | | | 15.72 | | | | | |  | | | 82.2% | | | | | | | | | | | |  | |  | | | |  |  |
|  |  | |  | | |  | | |  | | |  | | | |  | |  | | | | | | |  | | |  | | |  | | | | | | | |  |  | | | | | | | | | |  | |  | | |  | | | | | | | | | | | | |  |  | | | | | | | | |  | | |  | | | |  | |  |  |  |  |  |  |  |  |
| **Asthma** | 1.25 | | ( | | | 1.233 | | | - | | | 1.260 | | | | ) | |  | | | | | | | 0.72 | | | | ( | | | | | | 0.711 | | | | | | - | | 0.725 | | | | | | | | ) | | |  | | | | | | | | | | 0.92 | | | | | | ( | | | | | | | | | 0.911 | | | - | | 0.928 | | | | | | ) | | |  |  |
| **Back pain** | 4.55 | | ( | | | 4.478 | | | - | | | 4.626 | | | | ) | |  | | | | | | | 5.21 | | | | ( | | | | | | 5.110 | | | | | | - | | 5.307 | | | | | | | | ) | | |  | | | | | | | | | | 5.40 | | | | | | ( | | | | | | | | | 5.304 | | | - | | 5.487 | | | | | | ) | | |  |  |
| **Cancer** | 0.64 | | ( | | | 0.630 | | | - | | | 0.640 | | | | ) | |  | | | | | | | 0.81 | | | | ( | | | | | | 0.798 | | | | | | - | | 0.812 | | | | | | | | ) | | |  | | | | | | | | | | 0.85 | | | | | | ( | | | | | | | | | 0.838 | | | - | | 0.853 | | | | | | ) | | |  |  |
| **COPD** | 2.39 | | ( | | | 2.357 | | | - | | | 2.426 | | | | ) | |  | | | | | | | 2.57 | | | | ( | | | | | | 2.530 | | | | | | - | | 2.614 | | | | | | | | ) | | |  | | | | | | | | | | 2.12 | | | | | | ( | | | | | | | | | 2.092 | | | - | | 2.148 | | | | | | ) | | |  |  |
| **Diabetes** | 3.22 | | ( | | | 3.174 | | | - | | | 3.267 | | | | ) | |  | | | | | | | 3.62 | | | | ( | | | | | | 3.563 | | | | | | - | | 3.687 | | | | | | | | ) | | |  | | | | | | | | | | 4.76 | | | | | | ( | | | | | | | | | 4.678 | | | - | | 4.833 | | | | | | ) | | |  |  |
| **Heart disease** | 3.09 | | ( | | | 3.038 | | | - | | | 3.139 | | | | ) | |  | | | | | | | 3.09 | | | | ( | | | | | | 3.040 | | | | | | - | | 3.149 | | | | | | | | ) | | |  | | | | | | | | | | 3.74 | | | | | | ( | | | | | | | | | 3.680 | | | - | | 3.805 | | | | | | ) | | |  |  |
| **High cholesterol** | 4.01 | | ( | | | 3.943 | | | - | | | 4.076 | | | | ) | |  | | | | | | | 5.33 | | | | ( | | | | | | 5.226 | | | | | | - | | 5.428 | | | | | | | | ) | | |  | | | | | | | | | | 7.30 | | | | | | ( | | | | | | | | | 7.171 | | | - | | 7.431 | | | | | | ) | | |  |  |
| **Hypertension** | 7.14 | | ( | | | 7.012 | | | - | | | 7.271 | | | | ) | |  | | | | | | | 7.69 | | | | ( | | | | | | 7.533 | | | | | | - | | 7.850 | | | | | | | | ) | | |  | | | | | | | | | | 9.58 | | | | | | ( | | | | | | | | | 9.403 | | | - | | 9.757 | | | | | | ) | | |  |  |
| **Myocardium inf.** | 0.82 | | ( | | | 0.808 | | | - | | | 0.823 | | | | ) | |  | | | | | | | 0.61 | | | | ( | | | | | | 0.605 | | | | | | - | | 0.615 | | | | | | | | ) | | |  | | | | | | | | | | 0.41 | | | | | | ( | | | | | | | | | 0.412 | | | - | | 0.417 | | | | | | ) | | |  |  |
| **Stroke** | 0.47 | | ( | | | 0.465 | | | - | | | 0.472 | | | | ) | |  | | | | | | | 0.47 | | | | ( | | | | | | 0.468 | | | | | | - | | 0.477 | | | | | | | | ) | | |  | | | | | | | | | | 0.45 | | | | | | ( | | | | | | | | | 0.445 | | | - | | 0.451 | | | | | | ) | | |  |  |
| **At least one of the considered diseases** | 13.54 | | ( | | | 13.328 | | | - | | | 13.755 | | | | ) | |  | | | | | | | 14.08 | | | | ( | | | | | | 13.832 | | | | | | - | | 14.328 | | | | | | | | ) | | |  | | | | | | | | | | 15.72 | | | | | | ( | | | | | | | | | 15.499 | | | - | | 15.937 | | | | | | ) | | |  |  |
|  | **Women** | | | | | | | | | | | | | | | | | | | | | | | | | | | | | | | | | | | | | | | | | | | | | | | | | | | | | | | | | | | | | | | | | | | | | | | | | | | | | | | | | | | | | | | | | | | |  |  |
| **Year** | **2006** | | | | | | | | | | | | | | | | | | | | | |  | | | **2012** | | | | | | | | | | | | | | | | | | | | | | | | | | | | | | | | | | |  | | | | **2017** | | | | | | | | | | | | | | | | | | | | | | | | | | | |  |  |
| **Life expectancy 65+** | 21.67 | |  | | 100% | | | | | | | | | |  | | | | | | | |  | | | 22.43 | | | | | |  | 100% | | | | | | | | | | | | | | | |  | | | |  | | | |  | | | | | | | | | | 22.97 | | | | | | |  | | 100% | | | | | | | | | | | |  | | |  | | | |
| **HLE (disease-free)** | 3.69 | |  | | 17.0% | | | | | | | | | |  | | | | | | | |  | | | 3.93 | | | | | |  | 17.5% | | | | | | | | | | | | | | | |  | | | |  | | | |  | | | | | | | | | | 3.50 | | | | | | |  | | 15.2% | | | | | | | | | | | |  | | |  | | | |
| **LE with disease** | 17.97 | |  | | 83.0% | | | | | | | | | |  | | | | | | | |  | | | 18.50 | | | | | |  | 82.5% | | | | | | | | | | | | | | | |  | | | |  | | | |  | | | | | | | | | | 19.47 | | | | | | |  | | 84.8% | | | | | | | | | | | |  | | |  | | | |
|  |  | |  | |  | | |  | | |  | | | | | |  | | |  | |  | |  | | |  | | | | | | |  | |  | | | | | |  | | |  | | |  | | | | | | | | | | | |  | | |  | | | | | | | | | |  | | | |  | | | | | | | | | | | | | |  | | | |
| **Asthma** | | 1.41 | | ( | | | 1.402 | | | - | | | 1.422 | | | | | | ) | |  | | | 1.26 | | | | | | ( | | | | | | | 1.250 | | | | | | | - | | 1.269 | | | | | | | | | | ) | |  | | | | 1.49 | | | | | | | | | ( | | | | | | | | | 1.474 | | | - | | | 1.497 | | | | | | ) | |  |
| **Back pain** | | 9.92 | | ( | | | 9.787 | | | - | | | 10.061 | | | | | | ) | |  | | | 10.36 | | | | | | ( | | | | | | | 10.193 | | | | | | | - | | 10.529 | | | | | | | | | | ) | |  | | | | 11.03 | | | | | | | | | ( | | | | | | | | | 10.862 | | | - | | | 11.198 | | | | | | ) | |  |
| **Cancer** | | 0.45 | | ( | | | 0.443 | | | - | | | 0.447 | | | | | | ) | |  | | | 0.56 | | | | | | ( | | | | | | | 0.556 | | | | | | | - | | 0.562 | | | | | | | | | | ) | |  | | | | 0.85 | | | | | | | | | ( | | | | | | | | | 0.842 | | | - | | | 0.852 | | | | | | ) | |  |
| **COPD** | | 1.53 | | ( | | | 1.521 | | | - | | | 1.544 | | | | | | ) | |  | | | 1.66 | | | | | | ( | | | | | | | 1.643 | | | | | | | - | | 1.673 | | | | | | | | | | ) | |  | | | | 1.62 | | | | | | | | | ( | | | | | | | | | 1.604 | | | - | | | 1.629 | | | | | | ) | |  |
| **Diabetes** | | 3.72 | | ( | | | 3.675 | | | - | | | 3.756 | | | | | | ) | |  | | | 4.29 | | | | | | ( | | | | | | | 4.230 | | | | | | | - | | 4.342 | | | | | | | | | | ) | |  | | | | 4.96 | | | | | | | | | ( | | | | | | | | | 4.900 | | | - | | | 5.028 | | | | | | ) | |  |
| **Heart disease** | | 3.46 | | ( | | | 3.423 | | | - | | | 3.504 | | | | | | ) | |  | | | 3.75 | | | | | | ( | | | | | | | 3.693 | | | | | | | - | | 3.797 | | | | | | | | | | ) | |  | | | | 3.92 | | | | | | | | | ( | | | | | | | | | 3.866 | | | - | | | 3.969 | | | | | | ) | |  |
| **High cholesterol** | | 6.07 | | ( | | | 5.996 | | | - | | | 6.147 | | | | | | ) | |  | | | 7.51 | | | | | | ( | | | | | | | 7.397 | | | | | | | - | | 7.628 | | | | | | | | | | ) | |  | | | | 9.04 | | | | | | | | | ( | | | | | | | | | 8.908 | | | - | | | 9.178 | | | | | | ) | |  |
| **Hypertension** | | 11.35 | | ( | | | 11.190 | | | - | | | 11.508 | | | | | | ) | |  | | | 11.53 | | | | | | ( | | | | | | | 11.343 | | | | | | | - | | 11.719 | | | | | | | | | | ) | |  | | | | 12.30 | | | | | | | | | ( | | | | | | | | | 12.116 | | | - | | | 12.490 | | | | | | ) | |  |
| **Myocardium inf.** | | 0.48 | | ( | | | 0.481 | | | - | | | 0.485 | | | | | | ) | |  | | | 0.31 | | | | | | ( | | | | | | | 0.307 | | | | | | | - | | 0.309 | | | | | | | | | | ) | |  | | | | 0.40 | | | | | | | | | ( | | | | | | | | | 0.399 | | | - | | | 0.403 | | | | | | ) | |  |
| **Stroke** | | 0.47 | | ( | | | 0.471 | | | - | | | 0.476 | | | | | | ) | |  | | | 0.45 | | | | | | ( | | | | | | | 0.448 | | | | | | | - | | 0.453 | | | | | | | | | | ) | |  | | | | 0.46 | | | | | | | | | ( | | | | | | | | | 0.456 | | | - | | | 0.461 | | | | | | ) | |  |
| **At least one of the considered diseases** | | 17.97 | | ( | | | 17.784 | | | - | | | 18.166 | | | | | | ) | |  | | | 18.50 | | | | | | ( | | | | | | | 18.270 | | | | | | | - | | 18.725 | | | | | | | | | | ) | |  | | | | 19.47 | | | | | | | | | ( | | | | | | | | | 19.263 | | | - | | | 19.678 | | | | | | ) | |  |

Source: Authors’ calculations.
