# Supplementary material for: Trends in disease-free life expectancy at age 65 in Spain: Diverging patterns by sex, region and disease
Source: PLoS One. 2020 Nov 11;15(11):e0240923. doi: 10.1371/journal.pone.0240923 (PMC7657566; doi:10.1371/journal.pone.0240923)
Supplement: S2 Table — (DOCX) [file pone.0240923.s002.docx]

Table S2. Percentage of life expectancy with each disease at age 65 by sex in Spanish Autonomous Communities 2006, 2012 and 2017.

|  | **Autonomous Communities** | **Hypertension** | | |  | **Back Pain** | | |  | **High cholesterol** | | |  | **Diabetes** | | |  | **CVD** | | |  | **Respiratory** | | |
| --- | --- | --- | --- | --- | --- | --- | --- | --- | --- | --- | --- | --- | --- | --- | --- | --- | --- | --- | --- | --- | --- | --- | --- | --- |
| **Men** | | **2006** | **2012** | **2017** |  | **2006** | **2012** | **2017** |  | **2006** | **2012** | **2017** |  | **2006** | **2012** | **2017** |  | **2006** | **2012** | **2017** |  | **2006** | **2012** | **2017** |
|  | Andalusia | 42.91 | 39.22 | 46.39 |  | 31.54 | 32.20 | 31.91 |  | 25.96 | 33.14 | 30.40 |  | 22.93 | 26.13 | 27.83 |  | 19.13 | 21.66 | 21.32 |  | 17.25 | 14.79 | 16.93 |
|  | Aragon | 39.84 | 41.97 | 51.02 |  | 20.99 | 21.00 | 20.37 |  | 17.86 | 27.35 | 34.84 |  | 10.57 | 16.29 | 26.55 |  | 10.16 | 18.11 | 15.02 |  | 9.51 | 17.92 | 16.44 |
|  | Asturias | 39.04 | 36.85 | 49.25 |  | 34.01 | 23.42 | 44.63 |  | 17.40 | 22.17 | 32.66 |  | 14.00 | 16.71 | 15.93 |  | 13.80 | 29.03 | 28.56 |  | 27.22 | 14.43 | 18.13 |
|  | Balearic Islands | 40.30 | 43.99 | 43.29 |  | 26.70 | 28.04 | 30.08 |  | 24.85 | 19.98 | 25.65 |  | 17.75 | 16.59 | 21.44 |  | 26.08 | 4.60 | 26.79 |  | 15.76 | 19.08 | 14.29 |
|  | Canary Islands | 54.50 | 46.41 | 44.18 |  | 23.03 | 33.85 | 26.03 |  | 14.53 | 21.80 | 43.62 |  | 11.90 | 21.16 | 35.24 |  | 22.33 | 6.77 | 26.46 |  | 14.09 | 6.33 | 4.88 |
|  | Cantabria | 37.50 | 46.37 | 57.38 |  | 12.16 | 9.15 | 19.02 |  | 17.26 | 28.25 | 28.71 |  | 15.06 | 11.37 | 14.63 |  | 21.37 | 14.25 | 20.80 |  | 12.75 | 5.29 | 13.28 |
|  | Castile-León | 38.58 | 46.91 | 42.23 |  | 18.26 | 32.55 | 24.01 |  | 19.48 | 32.64 | 33.48 |  | 20.37 | 15.58 | 20.40 |  | 20.88 | 19.05 | 15.04 |  | 14.98 | 13.53 | 18.86 |
|  | Castile-La Manche | 38.40 | 40.78 | 56.29 |  | 29.55 | 20.84 | 21.34 |  | 25.76 | 25.29 | 41.42 |  | 17.10 | 13.88 | 30.43 |  | 15.33 | 15.08 | 24.18 |  | 10.87 | 15.60 | 18.09 |
|  | Catalonia | 36.11 | 55.23 | 47.86 |  | 26.80 | 22.74 | 31.91 |  | 21.46 | 33.33 | 40.83 |  | 14.04 | 18.74 | 21.34 |  | 18.33 | 21.91 | 20.28 |  | 20.83 | 20.45 | 9.57 |
|  | Valencia | 35.88 | 35.01 | 54.59 |  | 20.94 | 22.11 | 24.90 |  | 24.72 | 29.02 | 30.53 |  | 24.00 | 17.50 | 25.21 |  | 14.88 | 23.02 | 22.70 |  | 14.84 | 16.05 | 15.22 |
|  | Extremadura | 46.17 | 31.89 | 50.21 |  | 27.54 | 28.93 | 26.04 |  | 23.14 | 30.13 | 46.71 |  | 21.39 | 19.99 | 32.11 |  | 24.34 | 22.11 | 14.80 |  | 9.48 | 21.97 | 11.43 |
|  | Galicia | 38.18 | 42.72 | 56.52 |  | 34.90 | 33.79 | 38.19 |  | 26.35 | 29.10 | 48.91 |  | 18.32 | 20.38 | 25.27 |  | 25.18 | 20.27 | 26.27 |  | 21.67 | 13.42 | 18.42 |
|  | Madrid | 44.90 | 32.33 | 52.65 |  | 20.55 | 32.33 | 22.89 |  | 28.27 | 24.98 | 46.69 |  | 16.60 | 19.28 | 26.75 |  | 18.75 | 6.43 | 19.07 |  | 9.80 | 12.07 | 7.70 |
|  | Murcia | 43.17 | 46.76 | 58.62 |  | 32.85 | 35.52 | 15.25 |  | 13.98 | 35.01 | 42.49 |  | 17.30 | 25.35 | 20.08 |  | 27.02 | 17.14 | 19.23 |  | 14.94 | 21.74 | 16.15 |
|  | Navarre | 33.89 | 39.59 | 55.35 |  | 33.07 | 26.17 | 25.79 |  | 16.05 | 20.99 | 42.24 |  | 18.65 | 18.82 | 28.41 |  | 27.59 | 24.11 | 32.13 |  | 15.65 | 17.70 | 12.57 |
|  | Basque country | 41.27 | 35.01 | 50.24 |  | 22.02 | 38.02 | 31.38 |  | 18.83 | 20.36 | 43.58 |  | 20.11 | 22.85 | 23.37 |  | 18.83 | 19.48 | 20.41 |  | 16.39 | 9.97 | 11.85 |
|  | La Rioja | 45.26 | 34.45 | 32.66 |  | 24.24 | 24.81 | 23.65 |  | 9.47 | 20.85 | 32.92 |  | 16.25 | 17.19 | 15.44 |  | 11.48 | 12.71 | 12.22 |  | 5.75 | 10.76 | 10.11 |
| **Median** | | 39.84 | 40.78 | 50.24 |  | 26.70 | 28.04 | 25.79 |  | 19.48 | 27.35 | 40.83 |  | 17.30 | 18.74 | 25.21 |  | 19.13 | 19.05 | 20.80 |  | 14.94 | 14.79 | 14.29 |
|  |  |  |  |  |  |  |  |  |  |  |  |  |  |  |  |  |  |  |  |  |  |  |  |  |
|  |  |  |  |  |  |  |  |  |  |  |  |  |  |  |  |  |  |  |  |  |  |  |  |  |
| **Women** | |  |  |  |  |  |  |  |  |  |  |  |  |  |  |  |  |  |  |  |  |  |  |  |
|  | Andalusia | 51.77 | 58.00 | 53.00 |  | 49.46 | 55.76 | 52.82 |  | 26.40 | 34.06 | 35.35 |  | 23.27 | 23.24 | 28.90 |  | 18.97 | 24.45 | 18.92 |  | 8.53 | 12.52 | 12.01 |
|  | Aragon | 56.34 | 58.37 | 58.45 |  | 41.24 | 37.86 | 37.37 |  | 26.78 | 30.65 | 34.74 |  | 15.34 | 10.54 | 23.27 |  | 14.99 | 19.56 | 13.52 |  | 12.04 | 10.80 | 11.14 |
|  | Asturias | 57.51 | 54.17 | 51.91 |  | 53.47 | 54.59 | 66.50 |  | 28.37 | 22.73 | 35.60 |  | 12.64 | 13.61 | 22.40 |  | 18.47 | 20.36 | 26.61 |  | 11.78 | 10.36 | 12.90 |
|  | Balearic Islands | 44.18 | 35.87 | 30.47 |  | 39.88 | 49.64 | 54.04 |  | 25.33 | 24.89 | 28.12 |  | 19.49 | 19.36 | 15.38 |  | 18.75 | 11.14 | 15.65 |  | 13.60 | 10.46 | 13.11 |
|  | Canary Islands | 58.12 | 57.20 | 55.64 |  | 61.42 | 61.17 | 42.61 |  | 32.66 | 39.98 | 41.89 |  | 17.24 | 24.49 | 31.27 |  | 20.45 | 24.99 | 22.84 |  | 11.82 | 10.22 | 11.38 |
|  | Cantabria | 50.02 | 54.39 | 55.80 |  | 25.27 | 23.89 | 44.01 |  | 27.48 | 27.01 | 41.49 |  | 17.45 | 16.79 | 11.76 |  | 17.12 | 17.26 | 16.28 |  | 15.11 | 10.42 | 11.19 |
|  | Castile-León | 53.89 | 53.95 | 41.93 |  | 36.47 | 47.16 | 39.98 |  | 27.46 | 38.07 | 38.25 |  | 15.15 | 17.78 | 14.62 |  | 15.28 | 15.28 | 11.80 |  | 6.91 | 10.39 | 10.96 |
|  | Castile-La Manche | 53.01 | 56.05 | 60.91 |  | 45.45 | 48.63 | 38.97 |  | 30.26 | 41.65 | 42.54 |  | 27.53 | 26.74 | 18.57 |  | 22.36 | 18.70 | 22.95 |  | 12.88 | 14.64 | 8.29 |
|  | Catalonia | 43.70 | 50.31 | 54.55 |  | 43.53 | 32.60 | 59.37 |  | 23.83 | 36.42 | 36.64 |  | 16.05 | 18.54 | 18.61 |  | 15.82 | 16.31 | 16.13 |  | 9.59 | 11.21 | 11.12 |
|  | Valencia | 45.87 | 44.61 | 54.64 |  | 46.73 | 49.60 | 35.45 |  | 27.32 | 37.41 | 40.87 |  | 14.95 | 21.21 | 21.44 |  | 14.30 | 14.25 | 18.00 |  | 14.17 | 6.80 | 5.86 |
|  | Extremadura | 68.46 | 52.31 | 53.77 |  | 45.41 | 47.70 | 44.31 |  | 29.66 | 40.12 | 45.91 |  | 24.67 | 24.37 | 29.13 |  | 19.07 | 23.45 | 20.78 |  | 9.08 | 10.56 | 11.45 |
|  | Galicia | 53.37 | 55.22 | 58.23 |  | 54.82 | 52.31 | 62.54 |  | 34.30 | 42.02 | 52.50 |  | 13.90 | 20.26 | 19.84 |  | 23.58 | 18.48 | 24.32 |  | 17.40 | 15.09 | 20.42 |
|  | Madrid | 59.10 | 44.94 | 57.39 |  | 47.46 | 43.62 | 47.79 |  | 27.63 | 21.08 | 40.08 |  | 13.89 | 13.20 | 22.57 |  | 12.73 | 13.16 | 19.47 |  | 7.43 | 6.84 | 8.71 |
|  | Murcia | 62.26 | 58.20 | 60.50 |  | 44.52 | 53.30 | 26.56 |  | 37.17 | 37.76 | 47.09 |  | 19.34 | 24.29 | 23.56 |  | 21.72 | 19.65 | 17.33 |  | 14.49 | 15.70 | 9.27 |
|  | Navarre | 56.72 | 45.87 | 46.84 |  | 41.89 | 25.04 | 46.13 |  | 31.47 | 23.24 | 48.07 |  | 14.83 | 16.44 | 15.49 |  | 23.11 | 17.27 | 20.95 |  | 13.26 | 5.27 | 11.60 |
|  | Basque country | 54.10 | 45.79 | 46.17 |  | 38.86 | 46.22 | 40.72 |  | 34.01 | 29.12 | 32.85 |  | 11.61 | 11.76 | 11.71 |  | 18.30 | 17.19 | 16.56 |  | 8.78 | 9.67 | 7.49 |
|  | La Rioja | 53.38 | 46.45 | 44.14 |  | 42.25 | 31.67 | 32.30 |  | 18.00 | 24.67 | 33.36 |  | 8.93 | 17.83 | 12.98 |  | 20.54 | 10.82 | 10.50 |  | 9.92 | 6.67 | 6.94 |
| **Median** | | 53.89 | 53.95 | 54.55 |  | 44.52 | 47.70 | 44.01 |  | 27.63 | 34.06 | 40.08 |  | 15.34 | 18.54 | 19.84 |  | 18.75 | 17.27 | 18.00 |  | 11.82 | 10.42 | 11.14 |

Source: Authors’ calculations.
