# Supplementary material for: Correction: Trends in disease-free life expectancy at age 65 in Spain: Diverging patterns by sex, region and disease
Source: PLoS One. 2021 Mar 19;16(3):e0249115. doi: 10.1371/journal.pone.0249115 (PMC7978350; doi:10.1371/journal.pone.0249115)
Supplement: S1 Table — (PDF) [file pone.0249115.s001.pdf]

Table S1. Life expectancy at age 65 and remaining years with each disease (95% confident intervals) by sex in Spain 2006, 2012 and 2017.

	Men					
**Year**	**2006**		**2012**		**2017**	
**Life expectancy 65+**	17.70	100%	18.51	100%	19.12	100%
**HLE (disease-free)**	4.16	23.5%	4.43	23.9%	3.41	17.8%
**LE with disease**	13.54	76.5%	14.08	76.1%	15.72	82.2%
**Asthma**	1.25	(1.233 - 1.260)	0.72	(0.711 - 0.725)	0.92	(0.911 - 0.928)
**Back pain**	4.55	(4.478 - 4.626)	5.21	(5.110 - 5.307)	5.40	(5.304 - 5.487)
**Cancer**	0.64	(0.630 - 0.640)	0.81	(0.798 - 0.812)	0.85	(0.838 - 0.853)
**COPD**	2.39	(2.357 - 2.426)	2.57	(2.530 - 2.614)	2.12	(2.092 - 2.148)
**Diabetes**	3.22	(3.174 - 3.267)	3.62	(3.563 - 3.687)	4.76	(4.678 - 4.833)
**Heart disease**	3.09	(3.038 - 3.139)	3.09	(3.040 - 3.149)	3.74	(3.680 - 3.805)
**High cholesterol**	4.01	(3.943 - 4.076)	5.33	(5.226 - 5.428)	7.30	(7.171 - 7.431)
**Hypertension**	7.14	(7.012 - 7.271)	7.69	(7.533 - 7.850)	9.58	(9.403 - 9.757)
**Myocardium inf.**	0.82	(0.808 - 0.823)	0.61	(0.605 - 0.615)	0.41	(0.412 - 0.417)
**Stroke**	0.47	(0.465 - 0.472)	0.47	(0.468 - 0.477)	0.45	(0.445 - 0.451)
**At least one of the considered diseases**	13.54	(13.328 - 13.755)	14.08	(13.832 - 14.328)	15.72	(15.499 - 15.937)

	Women					
**Year**	**2006**		**2012**		**2017**	
**Life expectancy 65+**	21.67	100%	22.43	100%	22.97	100%
**HLE (disease-free)**	3.69	17.0%	3.93	17.5%	3.50	15.2%
**LE with disease**	17.97	83.0%	18.50	82.5%	19.47	84.8%
**Asthma**	1.41	(1.402 - 1.422)	1.26	(1.250 - 1.269)	1.49	(1.474 - 1.497)
**Back pain**	9.92	(9.787 - 10.061)	10.36	(10.193 - 10.529)	11.03	(10.862 - 11.198)
**Cancer**	0.45	(0.443 - 0.447)	0.56	(0.556 - 0.562)	0.85	(0.842 - 0.852)
**COPD**	1.53	(1.521 - 1.544)	1.66	(1.643 - 1.673)	1.62	(1.604 - 1.629)
**Diabetes**	3.72	(3.675 - 3.756)	4.29	(4.230 - 4.342)	4.96	(4.900 - 5.028)
**Heart disease**	3.46	(3.423 - 3.504)	3.75	(3.693 - 3.797)	3.92	(3.866 - 3.969)
**High cholesterol**	6.07	(5.996 - 6.147)	7.51	(7.397 - 7.628)	9.04	(8.908 - 9.178)
**Hypertension**	11.35	(11.190 - 11.508)	11.53	(11.343 - 11.719)	12.30	(12.116 - 12.490)
**Myocardium inf.**	0.48	(0.481 - 0.485)	0.31	(0.307 - 0.309)	0.40	(0.399 - 0.403)
**Stroke**	0.47	(0.471 - 0.476)	0.45	(0.448 - 0.453)	0.46	(0.456 - 0.461)
**At least one of the considered diseases**	17.97	(17.784 - 18.166)	18.50	(18.270 - 18.725)	19.47	(19.263 - 19.678)

Source: Authors' calculations.
