# Supplementary material for: Correction: Trends in disease-free life expectancy at age 65 in Spain: Diverging patterns by sex, region and disease
Source: PLoS One. 2021 Mar 19;16(3):e0249115. doi: 10.1371/journal.pone.0249115 (PMC7978350; doi:10.1371/journal.pone.0249115)
Supplement: S2 Table — (PDF) [file pone.0249115.s002.pdf]

Table S2. Percentage of life expectancy with each disease at age 65 by sex in Spanish Autonomous Communities 2006, 2012 and 2017.

Autonomous Communities	Hypertension			Back Pain			High cholesterol			Diabetes			CVD			Respiratory		
**Men**	**2006**	**2012**	**2017**	**2006**	**2012**	**2017**	**2006**	**2012**	**2017**	**2006**	**2012**	**2017**	**2006**	**2012**	**2017**	**2006**	**2012**	**2017**
Andalusia	42.91	39.22	46.39	31.54	32.20	31.91	25.96	33.14	30.40	22.93	26.13	27.83	19.13	21.66	21.32	17.25	14.79	16.93
Aragon	39.84	41.97	51.02	20.99	21.00	20.37	17.86	27.35	34.84	10.57	16.29	26.55	10.16	18.11	15.02	9.51	17.92	16.44
Asturias	39.04	36.85	49.25	34.01	23.42	44.63	17.40	22.17	32.66	14.00	16.71	15.93	13.80	29.03	28.56	27.22	14.43	18.13
Balearic Islands	40.30	43.99	43.29	26.70	28.04	30.08	24.85	19.98	25.65	17.75	16.59	21.44	26.08	4.60	26.79	15.76	19.08	14.29
Canary Islands	54.50	46.41	44.18	23.03	33.85	26.03	14.53	21.80	43.62	11.90	21.16	35.24	22.33	6.77	26.46	14.09	6.33	4.88
Cantabria	37.50	46.37	57.38	12.16	9.15	19.02	17.26	28.25	28.71	15.06	11.37	14.63	21.37	14.25	20.80	12.75	5.29	13.28
Castile-León	38.58	46.91	42.23	18.26	32.55	24.01	19.48	32.64	33.48	20.37	15.58	20.40	20.88	19.05	15.04	14.98	13.53	18.86
Castile-La Manche	38.40	40.78	56.29	29.55	20.84	21.34	25.76	25.29	41.42	17.10	13.88	30.43	15.33	15.08	24.18	10.87	15.60	18.09
Catalonia	36.11	55.23	47.86	26.80	22.74	31.91	21.46	33.33	40.83	14.04	18.74	21.34	18.33	21.91	20.28	20.83	20.45	9.57
Valencia	35.88	35.01	54.59	20.94	22.11	24.90	24.72	29.02	30.53	24.00	17.50	25.21	14.88	23.02	22.70	14.84	16.05	15.22
Extremadura	46.17	31.89	50.21	27.54	28.93	26.04	23.14	30.13	46.71	21.39	19.99	32.11	24.34	22.11	14.80	9.48	21.97	11.43
Galicia	38.18	42.72	56.52	34.90	33.79	38.19	26.35	29.10	48.91	18.32	20.38	25.27	25.18	20.27	26.27	21.67	13.42	18.42
Madrid	44.90	32.33	52.65	20.55	32.33	22.89	28.27	24.98	46.69	16.60	19.28	26.75	18.75	6.43	19.07	9.80	12.07	7.70
Murcia	43.17	46.76	58.62	32.85	35.52	15.25	13.98	35.01	42.49	17.30	25.35	20.08	27.02	17.14	19.23	14.94	21.74	16.15
Navarre	33.89	39.59	55.35	33.07	26.17	25.79	16.05	20.99	42.24	18.65	18.82	28.41	27.59	24.11	32.13	15.65	17.70	12.57
Basque country	41.27	35.01	50.24	22.02	38.02	31.38	18.83	20.36	43.58	20.11	22.85	23.37	18.83	19.48	20.41	16.39	9.97	11.85
La Rioja	45.26	34.45	32.66	24.24	24.81	23.65	9.47	20.85	32.92	16.25	17.19	15.44	11.48	12.71	12.22	5.75	10.76	10.11
**Median**	39.84	40.78	50.24	26.70	28.04	25.79	19.48	27.35	40.83	17.30	18.74	25.21	19.13	19.05	20.80	14.94	14.79	14.29

**Women**																		
Andalusia	51.77	58.00	53.00	49.46	55.76	52.82	26.40	34.06	35.35	23.27	23.24	28.90	18.97	24.45	18.92	8.53	12.52	12.01
Aragon	56.34	58.37	58.45	41.24	37.86	37.37	26.78	30.65	34.74	15.34	10.54	23.27	14.99	19.56	13.52	12.04	10.80	11.14
Asturias	57.51	54.17	51.91	53.47	54.59	66.50	28.37	22.73	35.60	12.64	13.61	22.40	18.47	20.36	26.61	11.78	10.36	12.90
Balearic Islands	44.18	35.87	30.47	39.88	49.64	54.04	25.33	24.89	28.12	19.49	19.36	15.38	18.75	11.14	15.65	13.60	10.46	13.11
Canary Islands	58.12	57.20	55.64	61.42	61.17	42.61	32.66	39.98	41.89	17.24	24.49	31.27	20.45	24.99	22.84	11.82	10.22	11.38
Cantabria	50.02	54.39	55.80	25.27	23.89	44.01	27.48	27.01	41.49	17.45	16.79	11.76	17.12	17.26	16.28	15.11	10.42	11.19
Castile-León	53.89	53.95	41.93	36.47	47.16	39.98	27.46	38.07	38.25	15.15	17.78	14.62	15.28	15.28	11.80	6.91	10.39	10.96
Castile-La Manche	53.01	56.05	60.91	45.45	48.63	38.97	30.26	41.65	42.54	27.53	26.74	18.57	22.36	18.70	22.95	12.88	14.64	8.29
Catalonia	43.70	50.31	54.55	43.53	32.60	59.37	23.83	36.42	36.64	16.05	18.54	18.61	15.82	16.31	16.13	9.59	11.21	11.12
Valencia	45.87	44.61	54.64	46.73	49.60	35.45	27.32	37.41	40.87	14.95	21.21	21.44	14.30	14.25	18.00	14.17	6.80	5.86
Extremadura	68.46	52.31	53.77	45.41	47.70	44.31	29.66	40.12	45.91	24.67	24.37	29.13	19.07	23.45	20.78	9.08	10.56	11.45
Galicia	53.37	55.22	58.23	54.82	52.31	62.54	34.30	42.02	52.50	13.90	20.26	19.84	23.58	18.48	24.32	17.40	15.09	20.42
Madrid	59.10	44.94	57.39	47.46	43.62	47.79	27.63	21.08	40.08	13.89	13.20	22.57	12.73	13.16	19.47	7.43	6.84	8.71
Murcia	62.26	58.20	60.50	44.52	53.30	26.56	37.17	37.76	47.09	19.34	24.29	23.56	21.72	19.65	17.33	14.49	15.70	9.27
Navarre	56.72	45.87	46.84	41.89	25.04	46.13	31.47	23.24	48.07	14.83	16.44	15.49	23.11	17.27	20.95	13.26	5.27	11.60
Basque country	54.10	45.79	46.17	38.86	46.22	40.72	34.01	29.12	32.85	11.61	11.76	11.71	18.30	17.19	16.56	8.78	9.67	7.49
La Rioja	53.38	46.45	44.14	42.25	31.67	32.30	18.00	24.67	33.36	8.93	17.83	12.98	20.54	10.82	10.50	9.92	6.67	6.94
**Median**	53.89	53.95	54.55	44.52	47.70	44.01	27.63	34.06	40.08	15.34	18.54	19.84	18.75	17.27	18.00	11.82	10.42	11.14

Source: Authors' calculations.
